# Confusion in the monitoring of coagulation function in pregnant and neonate patients with severe disease: A case reports and brief literature review

**DOI:** 10.1097/MD.0000000000035997

**Published:** 2023-11-17

**Authors:** Yuefang Wang, Jiajing He, Xia Zhang, Ge Zhang

**Affiliations:** a Department of Laboratory Medicine, West China Second University Hospital, Sichuan University, Chengdu, People’s Republic of China; b Key Laboratory of Birth Defects and Related Diseases of Women and Children (Sichuan University), Ministry of Education, Chengdu, People’s Republic of China.

**Keywords:** amniotic fluid embolism, case report, neonates, pregnancy, thrombotic and hemorrhagic disorders, tranexamic acid

## Abstract

**Rationale:**

Different populations have their own unique physiological and pathological characteristics. However, in specialized maternal and child hospitals, there is currently a lack of standardized methods for assessing coagulation dysfunction, both domestically and internationally.

**Patient concerns:**

A 19-day-old neonate was transferred to neonatal intensive care unit with cyanosis, nasal bleeding for 6 hours, and a consciousness disorder for 5 hours. A 33-year-old woman presented with hydramnios and a 39 + 3week intrauterine pregnancy. All indicators before delivery were normal, but postpartum hemorrhage occurred after delivery.

**Diagnoses:**

We retrospectively analyzed 1 neonate with pulmonary hemorrhage accompanied by thrombocytopenia and 1 pregnant patient with amniotic fluid embolism.

**Interventions:**

The new coagulation indicators, such as thrombin-antithrombin complex, plasmin-alpha 2 antiplasmin complex, thrombomodulin, and tissue plasminogen activator-plasminogen activator inhibitor-1 complex, have been indicated to be valuable. In neonates, it is necessary to continuously monitor special items combined with specific therapeutic agents, such as tranexamic acid. In cases where postpartum hemorrhage occurs with low fibrinogen levels, it is essential to effectively identify patients with severe amniotic fluid embolism from a high incidence of specimen clotting.

**Outcomes:**

The neonate’s oxygen saturation stabilized, and after 5 days of treatment with low molecular weight heparin, thrombin-antithrombin complex and plasmin-alpha 2 antiplasmin complex returned to normal levels. The pregnant began to remove the remaining thrombus, the patient’s condition recovered, and she had a good prognosis.

**Lessons:**

For pregnant and neonatal critical illnesses, it is necessary to develop personalized coagulation monitoring programs that provide realistic and reasonable treatment recommendations. Such programs should consider the unique physiological and pathological characteristics of different populations to ensure effective management of critically ill patients.

## 1. Introduction

It has been reported that over 40% of patients with severe disease will develop coagulation dysfunction, which can result in bleeding adverse events, increased blood transfusions, and mortality rates that are more than 4 times higher.^[1,2]^ However, there is currently a lack of standardized methods for quickly and accurately assessing coagulation dysfunction in patients with severe disease. In 2022, China released the “Chinese Expert Consensus On Standardized Assessment of Coagulopathy In patients with Severe Diseases”.^[3]^ By implementing these standardized methods, health care professionals can provide more effective care for severe patients with coagulopathy.

However, there is a special group of patients who require specific attention: pregnant patients and neonates. These groups differ from the normal population in various ways. Neonates, especially premature babies, have an increased risk of bleeding or thrombotic complications due to an immature coagulation system. This risk is heightened when other problems make haemostasis difficult, such as when placing a catheter. Additionally, infants experience rapid changes in haemostasis.^[4,5]^ Normal pregnancy is considered a pro-thrombotic state. Currently, recognized reference values and cutoff values for each hypercoagulable state index are established in the normal population.^[6]^

So, the new coagulation indicators have been indicated to be valuable, but their application can be challenging in terms of when to start monitoring, how to interpret results, and how to integrate them with treatment plans. In this case report, we describe 2 cases, including a neonate with rapidly progressive pulmonary hemorrhage with thrombocytopenia and 1 patient with amniotic fluid embolism (AFE) who experienced bleeding from low fibrinogen (Fg) that occurred immediately postpartum. The publication of this case report was done with the informed consent of the infant’s parents and the pregnant woman involved in the cases.

## 2. Case presentation

### 2.1. Neonate

A 19-day-old neonate was transferred to the neonatal intensive care unit of our hospital with cyanosis, nasal bleeding for 6 hours, and a consciousness disorder for 5 hours. During hospitalization, the patient was diagnosed with pulmonary hemorrhage. The indicators are shown in Table 1. The platelet (PLT) count had decreased from 601 to 187 × 10^9^/L on the first day of admission, indicating a decrease rate of more than 50%. Fg increased from 178 to 231 mg/dL. If PLT consumption is caused by disseminated intravascular coagulation (DIC), it is accompanied by a loss of Fg. However, at this time, D-dimer (DD) and fibrin degradation products (FDPs) were not increased significantly, and antithrombin, also continued to increase, which was not consistent with the expectations. Because PLT was within the normal reference range with no historical reference on the first day of admission, the laboratory did not verify the condition of the sample. Indeed, when we reexamined the specimen of that day, it was found that a clot was present in the sample, which explained the discrepancy in platelet counts.

**Table 1:** The indicators obtained from external hospitals and at the first day of admission in neonate.

Date	TAT (ng/mL)	PIC (ug/mL)	TM (TU/mL)	t-PAIC (ng/mL)	Fg (mg/dL)	PT (s)	APTT (s)	WBC (10^9^/L)	PLT (10^9^/L)	CRP (mg/L)	DD (mg/L FEU)	FDPs (ug/mL)	AT3 (%)	Anti Xa (U/mL)
April 27, 2022 (external hospital)	/	/	/	/	178	18.9	78	28.9	601	<1	4.89	12.3	43.2	/
April 28, 2022 1 AM	39.2	10.7	30	13.7	231	11.3	28	21.2	187	<1	9.12	15.8	69	/
April 28, 2022 15 PM	19.2	>40.0	30.3	7.6	151	11.9	31.3	15.9	428	24.8	15.21	46.4	60	/
April 29, 2022 15 PM	106.6	4.1	20.6	6.5	265	10.2	28.8	11.6	326	45	2.37	9.2	53	/
April 30, 2022 10 AM	13	0.7	20.5	10.7	367	11.2	50.1	10.8	347	22.1	1.63	4.2	51	0.4
May 01, 2022 10 AM	/	/	/	/	/	/	/	8.4	366	16.1	/	/	/	/
May 05, 2022 10 AM	2	0.7	23.7	3.4	434	12.2	74.6	/	/	6.1	/	/	/	0.4

AT = antithrombin, CRP = C-reactive protein, DD = D-dimer, FDPs = fibrin degradation products, Fg = fibrinogen, PIC = plasmin-alpha 2 antiplasmin complex, PLT = platelet, TAT = thrombin-antithrombin complex, TM = thrombomodulin, t-PAIC = tissue plasminogen activator-plasminogen activator inhibitor-1 complex.

The neonate developed significant shortness of breath and dyspnea, and pneumothorax was observed on the same day. Closed chest drainage was performed, and coagulation function was continuously monitored. Fg decreased to 151 mg/dL, C-reactive protein (CRP) increased to 24.8 mg/L, and DD and FDPs increased significantly to 15.21 mg/L FEU and 46.4 µg/mL, respectively. The changes in thrombin-antithrombin complex (TAT), plasmin-alpha 2 antiplasmin complex (PIC), thrombomodulin (TM), and tissue plasminogen activator-plasminogen activator inhibitor-1 complex (t-PAIC) were as expected, and PIC increased from 10.7 to > 40 µg/mL, indicating hyperfibrinolysis, which also explained why the patient experienced a significant decrease in Fg and PLT and an increase in DD and FDPs in just 10 hours. To control further bleeding, it was recommended to use antifibrinolytic agents (TnxAc, tranexamic acid) to inhibit fibrinolysis.^[7]^ By the next day, all coagulation indicators seemed to have improved, indicating effective control of fibrinolysis. However, CRP increased from 24.8 to 45 mg/L, which was confusing. To further investigate the cause, TAT, PIC, TM, and t-PAIC were reviewed. TAT increased nearly 5 fold from 19.2 to 106.6 ng/mL, while PIC decreased tenfold to 4.1 µg/mL, indicating a transition from hyperfibrinolysis to a hypercoagulable state. Further follow-up history revealed that the neonate was not stable at approximately 12:00 AM on the same day, and chest closed drainage was performed again. The child had unstable oxygen saturation (PaO2 < 40 mm Hg, oxygenation index persisted at > 20) from 2:00 pm, and when intravenous access was established in the child, obvious hypercoagulable performance was observed. TnxAc was discontinued urgently, and low molecular weight heparin (LMWH) was considered when TAT was significantly higher than PIC. The antibiotic regimen was also improved to fight infection. The neonate’s oxygen saturation stabilized, and after 5 days of treatment with LMWH, TAT and PIC returned to normal levels.

### 2.2. Pregnant woman

A 33-year-old woman presented with hydramnios and a 39^+3^week intrauterine pregnancy. All indicators before delivery were normal, but postpartum hemorrhage occurred after delivery. The indicators are shown in Table 2. The patient’s preoperative coagulation function was normal, but bleeding was present from the perineal incision after delivery. At 11:42 am after delivery, coagulation indicators significantly changed with a decrease in Fg from 485 to < 50 mg/dL and prolonged PT/APTT. Large amounts of flocculent precipitates were visible in the plasma. According to clinical experience, we did not reject this specimen, and it is very likely that the patient was already in an ultra-high coagulation state, leading to the generation of nonhigh-quality specimens, so we chose to actively communicate with the clinic. Treatment with Fg and TnxAc was not effective in achieving haemostasis. Evaluation of TAT, PIC, TM, and t-PAIC were performed at 01:09 pm, with TAT being significantly increased to > 120 ng/mL and large amounts of flocculent precipitates also visible in the plasma, indicating that the purpura was due to the ultra-high hypercoagulable state. TM was within the normal range, indicating no systemic or extensive endothelial cell damage. PIC was significantly higher at > 40 µg/mL, indicating hyperfibrinolytic-type DIC with heavy consumption of Fg. Acute AFE was clinically suspected. At 2:02 pm, 600 mL plasma and 8 g Fg were infused again. At 2:41 pm, the maternal condition was stable, Fg increased from < 50 to 168 mg/dL, TAT significantly decreased from > 120 to 18 ng/mL, PIC was still high at > 40 µg/mL, and t-PAIC was increased to 11.1 ng/mL, suggesting that; In the absence of anticoagulant treatment, hypercoagulable factors no longer existed; The interaction between PIC and t-PAIC was normal at a higher level, the body began to remove the remaining thrombus, DD and FDPs were still at a higher level, and the body was basically in a stable state. At 9:02 pm, the patient’s condition recovered, and she had a good prognosis.

**Table 2:** The indicators of pregnant woman with AFE.

Date	TAT (ng/mL)	PIC (ug/mL)	TM (TU/mL)	t-PAIC (ng/mL)	Fg (mg/dL)	PT (s)	APTT (s)	DD (mg/L FEU)	FDPs (ug/mL)	AT3 (%)	Clinical symptom
8:28 AM	/	/	/	/	485	11.8	24,7	/	/	/	/
11:42 AM	/	/	/	/	<50	20.6	40.5	100.2	309.8	59	Postpartum perineal incision multiple bleeding is obvious
13:09 PM	>120	>40	13.1	6.7	<50	14	22	/	/	/	/
14:41 PM	18	>40	11.5	11.1	168	26.9	42	312.21	603.63	61	Stable
21:02 PM	38.5	19.2	13.1	18	259	14.5	34.1	/	/	/	Stable

AFE = amniotic fluid embolism, DD = D-dimer, FDPs = fibrin degradation products, Fg = fibrinogen, PIC = plasmin-alpha 2 antiplasmin complex, TAT = thrombin-antithrombin complex, TM = thrombomodulin, t-PAIC = tissue plasminogen activator-plasminogen activator inhibitor-1 complex.

The incidence of AFE is low, and clinical judgement may be delayed. Rejecting such specimens with cloud precipitation may not be appropriate in obstetrics, especially in emergency patients. DD and FDPs testing can be added to determine if the specimen clot or hypercoagulable state caused cloud precipitation. After reviewing the DD and FDPs data of the clot specimens in our laboratory (Table 3), which is different from the real ultra-high coagulation process in vivo, FDPs was often < 100 µg/mL, and DD was < 40 mg/L FEU. In this case, the DD was 100.2 mg/L FEU, the FDPs was 309.8 µg/mL, and cloud precipitation due to in vitro clots could be ruled out. TAT and PIC tests can also be added, and only a real hypercoagulable state in the body will lead to such a high index change. Low Fg levels in obstetric patients should raise suspicion for severe AFE. When AFE occurs, obtaining high-quality specimens can be difficult due to the highly active coagulation state in the body.

**Table 3:** DD and FDPs level of clot specimens in our laboratory.

	DD (mg/L FEU) (mg/L FEU)	FDPs (ug/mL) (ug/mL)
1	3.44	13.7
2	0.4	1.4
3	0.73	2.6
4	1.81	4.9
5	4.23	9.9
6	2.45	6.1
7	13.77	24.6
8	6.82	16.8
9	0.13	0.3
10	3.72	9.4
11	32.18	55.2
12	1.06	3
13	5.36	14.9
14	1.59	4.9
15	16.36	49.5
16	0.48	1.6
17	0.3	1.7
18	3.95	15
19	1.47	3.4
20	2.42	5.4
21	0.14	0.7
22	1.01	4.5
23	0.85	8.5
24	33.81	92.1
25	0.42	1.3
26	23.09	24.3
27	3.38	7.8
28	34.38	97.9
29	0.72	3.2
30	7.11	22.7
31	4.59	13
32	3.9	9.1
33	7.87	19
34	0.48	2
35	0.29	0.9
36	0.14	1.1
37	17.74	58.7
38	1.64	5.2
39	7.94	18.3
40	1.35	3.4

DD = D-dimer, FDPs = fibrin degradation products.

## 3. Discussion

### 3.1. Clinical manifestations for neonates with thrombocytopenia and developing thrombosis

In the neonatal intensive care unit setting, neonates with severe thrombocytopenia (PLT < 50 × 10^9^/L) are at an increased risk of developing thrombosis, with a reported risk between 2.4 and 5%.^[8]^ However, the PLT can be a challenging indicator for the difficulty in collecting blood from neonates due to their small-diameter blood vessels and poor peripheral circulation. In the case of the neonate transferred from an external hospital, the initial PLT count was within the normal reference range. The laboratory did not verify the condition of the sample and directly reviewed the report. However, clinicians were able to detect a significant decrease in PLT count, which allowed for the early detection of critical illness and effective assessment of the rapid development of the newborn.

If routine coagulation indicators are normal and antithrombin is reduced, the risk of thrombosis will increase, especially if there are other stimuli, such as surgical trauma. TAT, PIC, TM and t-PAI-C are suitable for early diagnosis and risk assessment of thrombosis in high-risk populations. TAT/DD should be dynamically monitored to adjust the intensity of anticoagulation therapy, or the patient should be switched from anticoagulation to haemostasis therapy. The newborn population is more sensitive to changes in CRP than adults, and a CRP level of > 10 mg/L can indicate neonatal inflammation. In this case, when the coagulation function gradually returned to normal, CRP increased significantly, which indicated the aggravation of the disease and indicated the initiation of new markers of coagulation.

### 3.2. Special coagulation indicators- how to integrate them with treatment plans in neonates

Data on different management strategies and the efficacy and safety of specific therapeutic agents in the neonatal population are limited. TnxAc is an antifibrinolytic agent that is effective in treating neonates, especially those with pulmonary hemorrhage,^[9]^ despite the lack of specific guidelines for its use in this population. However, its use should be closely monitored, as it can accelerate the formation of venous thromboembolism in hypercoagulable states. In this case, the patient’s TAT increased from 19.2 to 106.6 ng/mL within 1 day after the use of TnxAc, which is puzzling. Although previous concerns regarding the risk of thrombosis with TnxAc have been largely disproven, its thrombotic safety should still be considered. Previous concerns regarding risks of thrombosis have largely been disproven in randomized trials.^[10,11]^ In a systematic review and meta-analysis, the use of TnxAc reduced all-cause mortality without increasing the risk of thrombotic complications.^[12]^ In this case, does hypoxia caused by poor drainage of the drainage tube exacerbate thoracic hypoxia and cause endothelial cell damage leading to hypercoagulability? However, TM did not increase, so the rapid increase in TAT for a short period could not be explained, but with the late intensification of antibiotic combination therapy and anticoagulation with LMWH, TAT markedly reduced, which was possibly due to the exacerbation of infection.

### 3.3. Special coagulation indicators: how to identify flocculent precipitates in the ultra-high coagulation state

AFE is a rare (1 to 12 cases per 100,000 deliveries) but serious condition that can occur during labor or within 30 minutes postpartum.^[13,14]^ It has a high mortality rate and is usually characterized by a sudden, catastrophic, and rapidly progressive clinical presentation. DIC causes hemorrhage in over 80% of patients with AFE.^[10,11]^ The decrease in Fg levels observed in AFE cannot be reasonably explained by the size of the surgical wound or high demand for haemostasis; therefore, DIC testing should be performed to determine the cause of the decrease in Fg and rule out sample clots. TAT > 120 ng/mL, PIC > 40 µg/mL, elevated DD/FDPs, reduced PLT counts, and inability to perform effective haemostasis are typical manifestations of AFE,^[12,15]^ which is caused by the placental chorionic component entering the mother’s blood circulation, leading to hyperfibrinolytic DIC and a large loss of Fg.

The laboratory’s experience with the case of severe AFE highlights the importance of correctly and timely identifying such specimens to avoid delays in patient management. To exclude the possibility of specimen clot interference, it is recommended to perform additional tests such as DD/FDPs or TAT/PIC. It is important to note that samples from postpartum hemorrhage patients with low Fg should not be rejected, even if there are clots present. In such cases, obtaining high-quality samples can be difficult due to the patient’s hypercoagulable state.

## Acknowledgements

We thank AJE for editing the language of a draft of this manuscript.

## Author contributions

**Conceptualization:** Ge Zhang.

**Data curation:** Jiajing He.

**Formal analysis:** Jiajing He.

**Investigation:** Yuefang Wang.

**Methodology:** Yuefang Wang.

**Software:** Jiajing He.

**Writing – original draft:** Yuefang Wang.

**Writing – review & editing:** Xia Zhang.
